# Harnessing the versatility of hydrazones through electrosynthetic oxidative transformations

**DOI:** 10.3762/bjoc.20.175

**Published:** 2024-08-14

**Authors:** Aurélie Claraz

**Affiliations:** 1 Institut de Chimie des Substances Naturelles, CNRS, Univ. Paris-Saclay, 1 Avenue de la Terrasse, 91198 Gif-sur-Yvette Cedex, France

**Keywords:** C–H functionalization, diazo compound, electrosynthesis, hydrazone, nitrogen-containing heterocycle

## Abstract

Hydrazones are important structural motifs in organic synthesis, providing a useful molecular platform for the construction of valuable compounds. Electrooxidative transformations of hydrazones constitute an attractive opportunity to take advantage of the versatility of these reagents. By directly harnessing the electrical current to perform the oxidative process, a large panel of organic molecules can be accessed from readily available hydrazones under mild, safe and oxidant-free reaction conditions. This review presents a comprehensive overview of oxidative electrosynthetic transformations of hydrazones. It includes the construction of azacycles, the C(sp^2^)−H functionalization of aldehyde-derived hydrazones and the access to diazo compounds as either synthetic intermediates or products. A special attention is paid to the reaction mechanism with the aim to encourage further development in this field.

## Introduction

Hydrazones represent an important class of organic compounds. They can be readily prepared through the condensation of hydrazine derivatives with aldehydes or ketones. They have found widespread applications in materials sciences and supramolecular chemistry [1–5]. Importantly, they are versatile reagents in organic synthesis. They have for instance been frequently employed for the construction of azacycles through various cyclization protocols or cycloaddition reactions [6–10]. Early work in this field includes the well-known Fischer indole synthesis [11]. Additionally, they have been harnessed as valuable intermediates in Wolff–Kishner reduction reactions [12–14] or for the synthesis of various olefins via diazo or vinyllithium intermediates in the Bamford–Stevens reaction [15] and Shapiro reaction [16], respectively [17]. SAMP/RAMP ((*S*)/(*R*)-1-amino-2-methoxymethylpyrrolidine)hydrazones could be also key intermediates for the asymmetric synthesis of α-substituted aldehydes and ketones [18–19]. Interestingly, depending on the substitution pattern, the C=N bond can feature different electronic properties [20]. For instance, various hydrazones have been employed for the asymmetric preparation of chiral amines through the addition of nucleophilic partners [21–22] while the azaenamine character of some aldehyde-derived hydrazones has been demonstrated in the coupling with suitable electrophiles such as Michael acceptors [23–24]. Last but not least, the C=N bond of hydrazones can act as radical acceptors for the synthesis of functionalized amines or hydrazones through reductive functionalization [21,25–26] or oxidative C(sp^2^)–H functionalization [27–28], respectively. Consequently, given their rich reactivity profile, exploring new synthetic transformations of hydrazones is of significant importance and can contribute to the formation of novel organic compounds.

Electrosynthesis enables the generation of either radical, radical ionic or ionic species [29] under mild and environmentally friendly reaction conditions [30–31]. The direct use of electrical current to drive oxidative and reductive processes precludes the reliance on toxic or dangerous redox reagents [32]. The explosive renewal of interest in this technology and the resulting recent achievements have brought it at the forefront of organic synthesis. Electrooxidative transformations of hydrazones offer appealing opportunities to take advantage of the versatility of this reagent. Such an approach can either ameliorate the previous methods in a more sustainable and efficient fashion or provide a mean for the discovery of new reactivity. Herein, this review aims to give an overview of the state of the art in the electrochemical oxidative transformations of hydrazones. It is organized in four main parts: (i) synthesis of azacycles, (ii) synthesis of functionalized hydrazones through C(sp^2^)–H functionalization of aldehyde-derived hydrazones, (iii) access to diazo compounds, and (iv) synthesis of miscellaneous compounds. For reactions carried out under constant current electrolysis, the reported current applied (in A or mA) is depicted. Additionally, when possible, and for better accuracy, the current density (in mA·cm^−2^) has been calculated based on the size of the electrode portion immersed in the solution, as described in the experimental section. This information is provided in brackets.

## Review

### Synthesis of azacycles

Nitrogen-containing heterocyclic compounds possess numerous applications in various domains such as materials science, agrochemistry and medicinal chemistry. Especially, 60% of all FDA-approved drugs in the United States contain at least one azacycle [33]. Therefore, the development of gentle and efficient methods for accessing these heterocycles is an ongoing pursuit for synthetic chemists. As mentioned above, with their two nitrogen atoms, hydrazones constitute unique synthons for constructing azacycles. Resorting to electrochemical approaches furnishes original compounds. Although the first report dates back to 1974, this tool has only been seriously considered in the last 15 years.

#### Oxidative cyclization of hydrazones

**Scheme 1:**
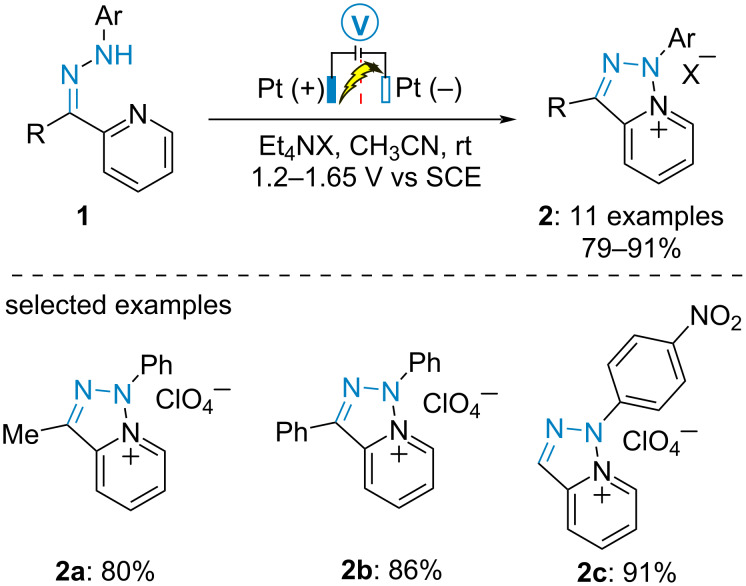
Synthesis of triazolopyridinium salts [34–36].

In 1974, Tabaković et al. published the oxidative cyclization of 2-acetylpyridine-derived *N*-phenylhydrazone **1a** to form triazolopyridinium salt **2a** [34]. The process was further applied to various 2-acetylpyridine and 2-benzoylpyridine derivatives (Scheme 1) [35–36]. The corresponding pyridinium salts **2** were obtained in high yields when the electrolysis was conducted in a divided cell under potentiostatic conditions at 1.2–1.65 V vs SCE in acetonitrile.

**Scheme 2:**
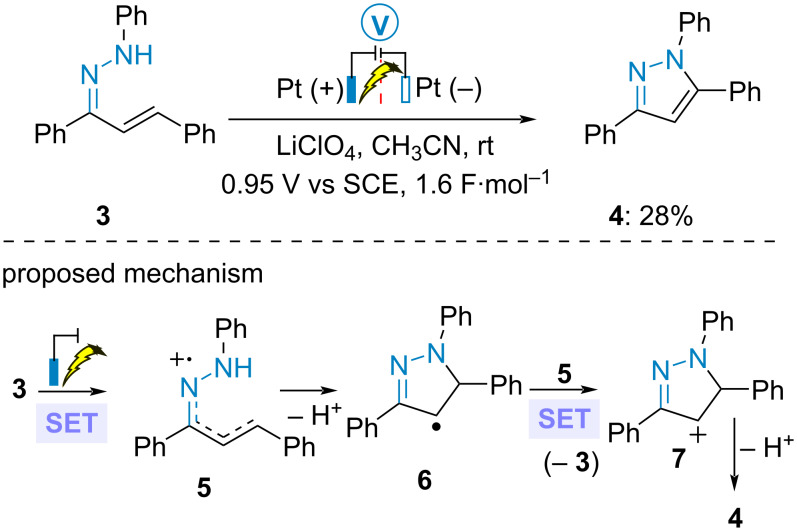
Synthesis of pyrazoles [37].

In 1976, the same group reported the anodic cyclization of chalcone-derived *N*-phenylhydrazone **3** to pyrazole **4** in a divided cell at constant current (Scheme 2) [37]. The obtained poor yield was explained by the formation of dimeric side products. Cyclic voltammetry analysis suggested an initial anodic single electron transfer (SET) to radical cation **5**, cyclization and deprotonation. Subsequent SET oxidation in solution by **5** led to cation **7**. Final deprotonation furnished aromatic cycle **4**.

**Scheme 3:**
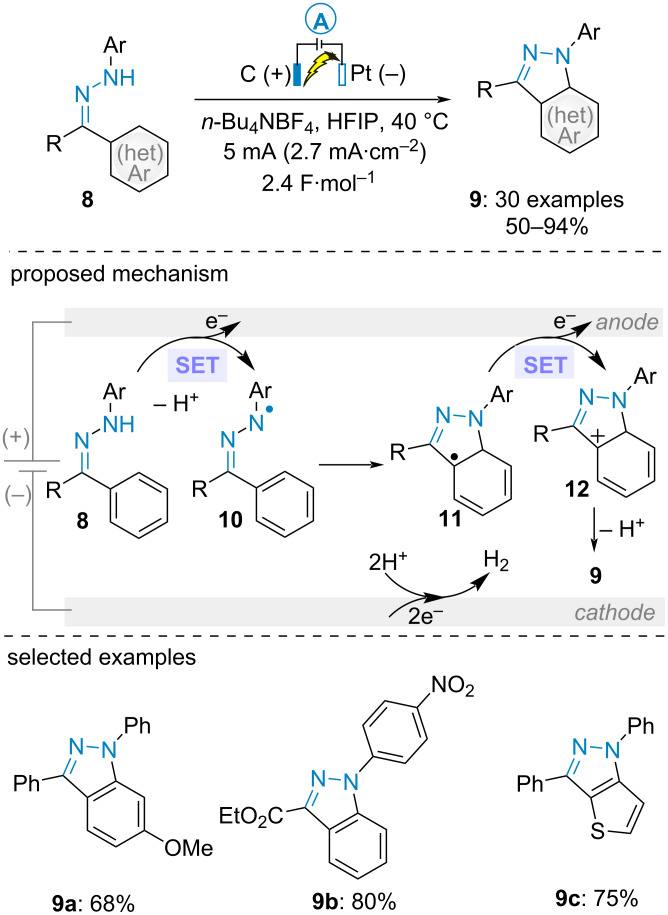
Synthesis of indazoles from ketone-derived hydrazones [38].

In 2022, Zhang et al. documented the synthesis of 1*H*-indazoles **9** via the electrooxidative cyclization of (hetero)aromatic ketones-derived *N*-phenylhydrazones **8** (Scheme 3) [38]. In contrast with the above-mentioned works, the electrolysis was carried out in an undivided cell under galvanostatic conditions. High yields were obtained regardless of the electronic properties of the substituents on the *N*-phenyl ring. When dissymmetric diaryl ketone-derived substrates were employed, the C–N bond formation occurred selectively on the most electron rich aromatic ring. According to the proposed mechanism, this dehydrogenative cyclization of hydrazones initiated with the SET anodic oxidation of the hydrazone and deprotonation to form the *N*-centered radical **10**. After aza-cyclization on the aromatic ring, a second SET oxidation and deprotonation delivered the heterocycle **9**. This mechanism was supported by cyclic voltammetry analysis of a model substrate (1-(diphenylmethylene)-2-(4-nitrophenyl)hydrazine), which displayed three oxidation peaks (0.9, 1.7 and 2.2 V vs Ag^+^/Ag in HFIP ). The authors assumed that the two first peaks would correspond to the oxidation of **8** to **10** and **11** to **12** and that the oxidation of **10** would be responsible for the final one, which is consistent with the finding of ketone side-product.

**Scheme 4:**
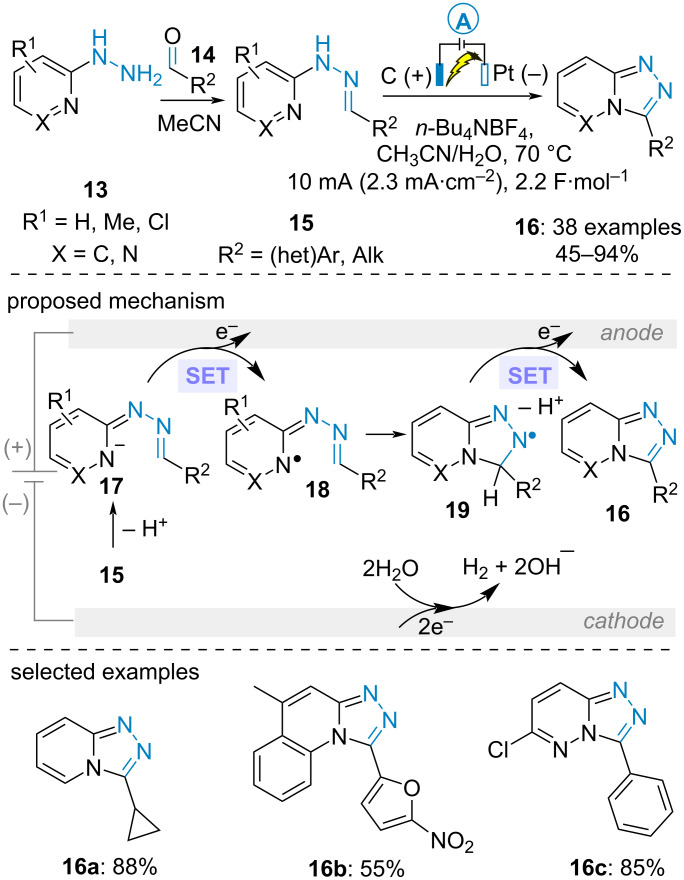
Intramolecular C(sp^2^)–H functionalization of aldehyde-derived *N*-(2-pyridinyl)hydrazones for the synthesis of 1,2,4-triazolo[4,3-*a*]pyridines [39].

In 2018, the group of Zhang established an intramolecular C(sp^2^)–H functionalization of aldehyde-derived *N-(*2-pyridinyl)hydrazones **15** to produce 1,2,4-triazolo[4,3-*a*]pyridines **16** (Scheme 4) [39]. Interestingly, the hydrazone was in situ prepared prior to the electrolysis through the condensation of 2-hydrazinopyridine **13** and various aromatic, aliphatic and α,β-unsaturated aldehydes **14**. The electrooxidative transformation was performed under constant current at 7 mA in a mixture of acetonitrile and water under heating. From a mechanistic point of view, the authors proposed the formation of *N*-pyridyl radical **18** through the anodic oxidation of in situ-generated anion **17**. Subsequent radical cyclization, second anodic cyclization and deprotonation yielded the fused heterocycle **16**.

**Scheme 5:**
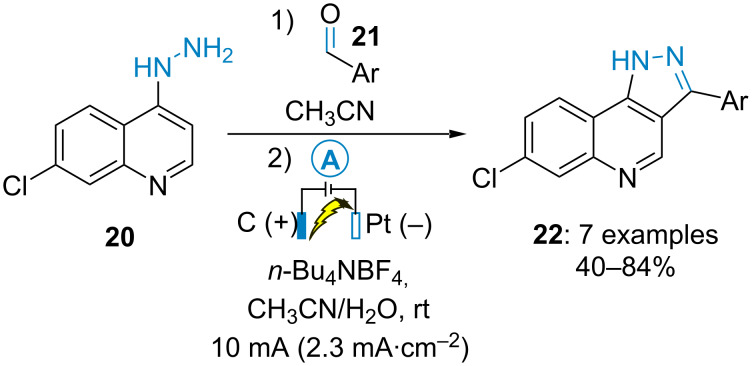
Synthesis of pyrazolo[4,3-*c*]quinoline derivatives [40].

Similarly, Youssef and Alajimi disclosed the electrochemical synthesis of pyrazolo[4,3-*c*]quinoline derivatives **22** from 7-chloro-4-hydrazinoquinolines **20** and aromatic aldehydes **21** (Scheme 5) [40].

**Scheme 6:**
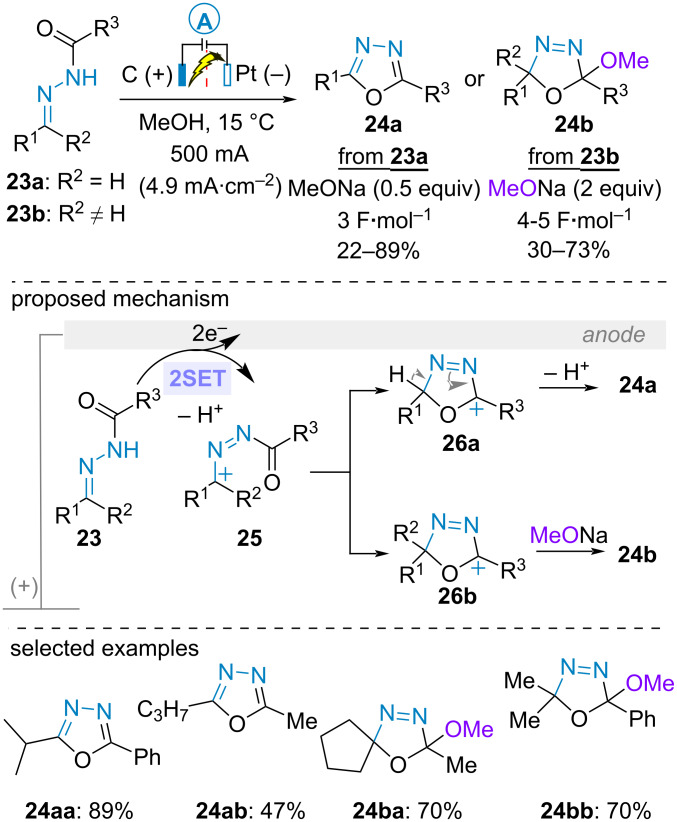
Synthesis of 1,3,4-oxadiazoles and Δ^3^-1,3,4-oxadiazolines [41].

In 1992, Chiba and Okimoto reported the electrooxidative cyclization of aldehyde and ketone-derived *N*-acylhydrazones **23a** and **23b** to build 1,3,4-oxadiazoles **24a** and Δ^3^-l,3,4-oxadiazolines **24b**, respectively. Using sodium methoxide as basic supporting electrolyte in a divided cell equipped with carbon rod anodes and a platinium coil cathode, the transformation would involve cyclization of carbocationic species **25** to form the intermediate **26**. From **23a** (R^2^ = H), the latter would undergo deprotonation delivering the oxadiazole **24a**. Alternatively, from **23b** (R^2^ ≠ H), nucleophilic addition of methanol to oxycarbenium **26b** yielded the oxadiazoline **24b** (Scheme 6) [41–42].

**Scheme 7:**
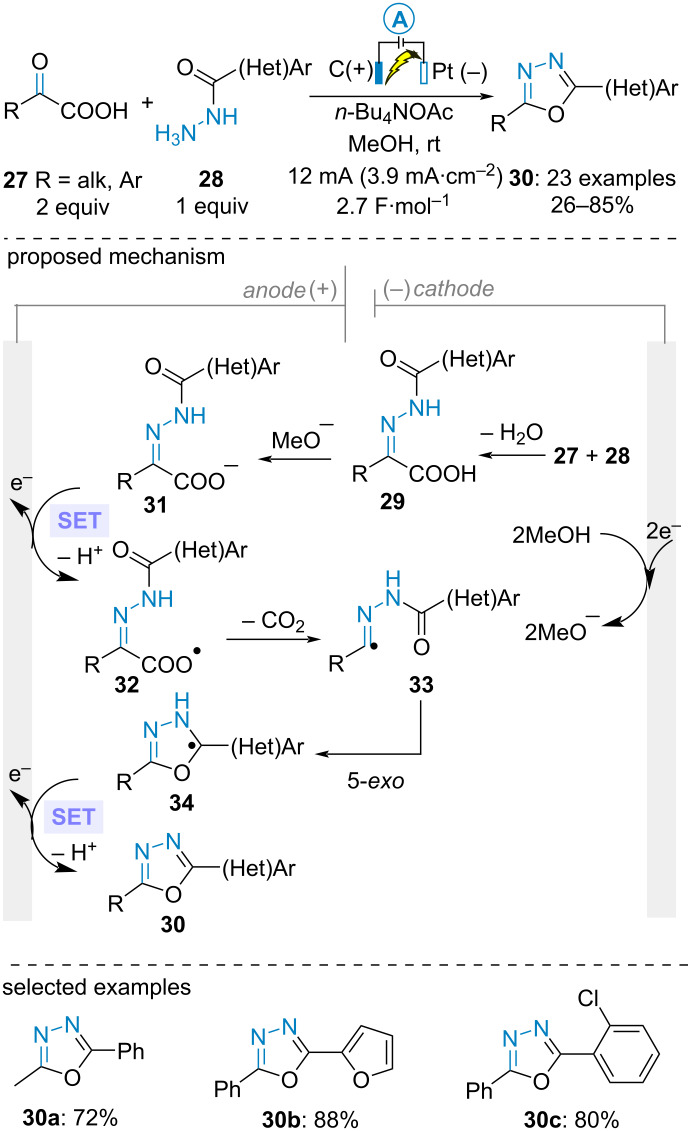
Synthesis of 1,3,4-oxadiazoles [43].

In 2020, Lei, Zhang and Gao et al. described the electrooxidative cyclization of in situ-generated α-keto acid-derived *NH*-acylhydrazones to build 1,3,4-oxadiazole derivatives in good yields. The electrolysis was conducted under galvanostatic conditions in methanol using a carbon graphite anode and a platinum cathode. From a mechanistic point of view, in situ condensation of acylhydrazines **28** with α-keto acids **27** produced hydrazones **29**. After deprotonation, the authors proposed that the carboxylate anion underwent SET anodic oxidation/decarboxylation/radical cyclization sequence to form radical intermediates **34**. Subsequent second anodic oxidation and deprotonation yielded the desired heteroaromatic 5-membered rings **30**. Importantly, a control experiment using benzaldehyde-derived *N*-benzoylhydrazone delivered the corresponding 1,3,4-oxadiazole in only 25% yield. This result indicates that benzaldehyde-derived hydrazone is a less probable reaction intermediate, thereby supporting the proposed mechanism (Scheme 7) [43].

**Scheme 8:**
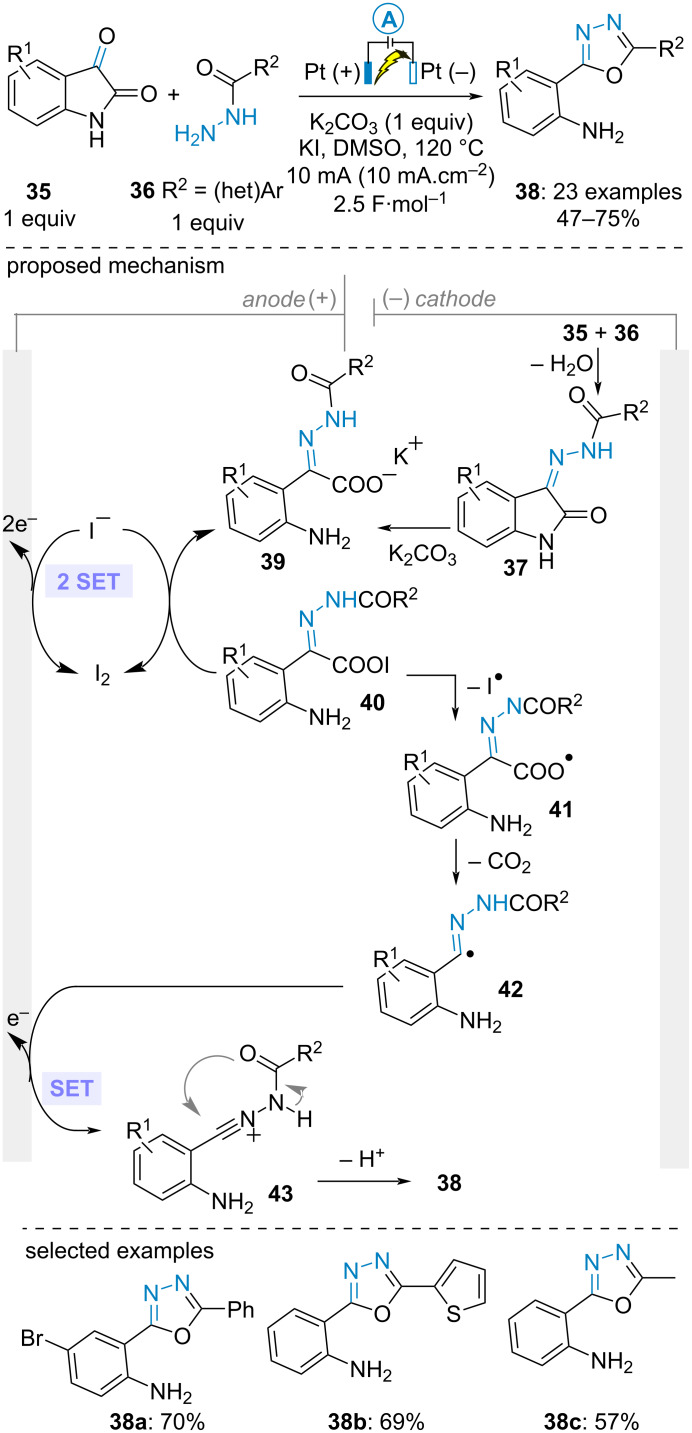
Synthesis of 2-(1,3,4-oxadiazol-2-yl)anilines [44].

In parallel, Sheng and Zhang et al. found that 2-(1,3,4-oxadiazol-2-yl)aniline derivatives **38** could be electrochemically synthesized from isatins **35** and acylhydrazine **36**. The transformation was carried out in an undivided cell at high temperature in DMSO using potassium iodide as supporting electrolyte with potassium carbonate as a base and two platinum electrodes. Mechanistically, condensation of the two reactants formed isatin-derived acylhydrazone **37**. Hydrolysis of the latter in the presence of the inorganic base gave rise to potassium carboxylate **39**. Meanwhile, two consecutive SET oxidations of the iodide anion generated molecular iodine which reacted with **39** to furnish unstable hypoiodous anhydride **40**, thereby triggering the key CO_2_ extrusion. The resulting C(sp^2^)-centered radical **42** underwent a SET anodic oxidation/cyclization/deprotonation sequence to yield the oxadiazole derivative **38**. As such, the iodide electrolyte served as an electromediator to both promote the decarboxylation process and protect the aniline product from overoxidation. Importantly, a control experiment without electricity but in the presence of molecular iodine instead proceeded smoothly, thereby confirming the critical role of in situ-generated molecular iodine (Scheme 8) [44].

#### Formal cycloaddition

Hydrazones constitute a versatile building block for the construction of azacycles through formal cycloaddition reactions. Under oxidative electrochemical conditions, either the oxidation of the hydrazone or the partner might be investigated offering numerous possibilities for the assembly of heterocycles.

**Scheme 9:**
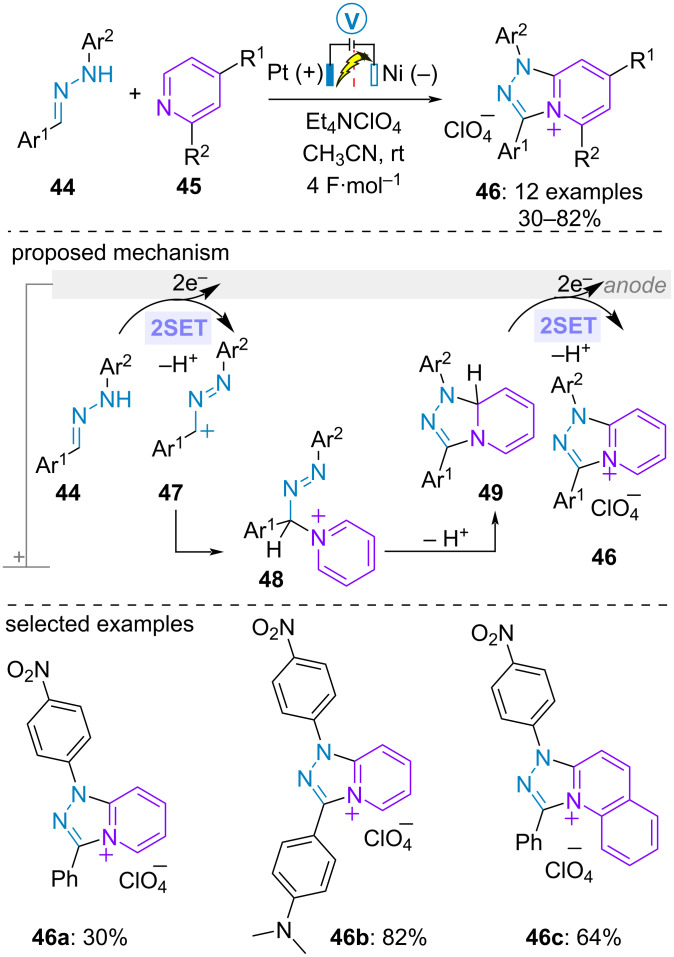
Synthesis of fused *s*-triazolo perchlorates [45].

As early as 1988, Tabaković and Gunić examined the anodic oxidation of aldehyde-derived *NH*-arylhydrazones **44** in the presence of pyridine and (iso)quinolone derivatives **45** in acetonitrile-tetraethylammonium perchlorate solution under constant potential in a divided cell equipped with platinum gauze anode and nickel cathode. Such a transformation constituted a straightforward route to the corresponding fused *s*-triazolo perchlorates **46** in moderate to high yield. Coulometric analysis established that the electrolysis was a four-electron oxidative process. Based on this study, the authors proposed the initial anodic oxidation of hydrazone **44** through the loss of two electrons and one proton to form cation **47**. Subsequent nucleophilic addition of the azaarene led to new highly acidic cationic species **48**. The latter underwent deprotonation and cyclization to form fused system **49**. Final two-electron oxidation and deprotonation delivered the ionic product **46** (Scheme 9) [45].

**Scheme 10:**
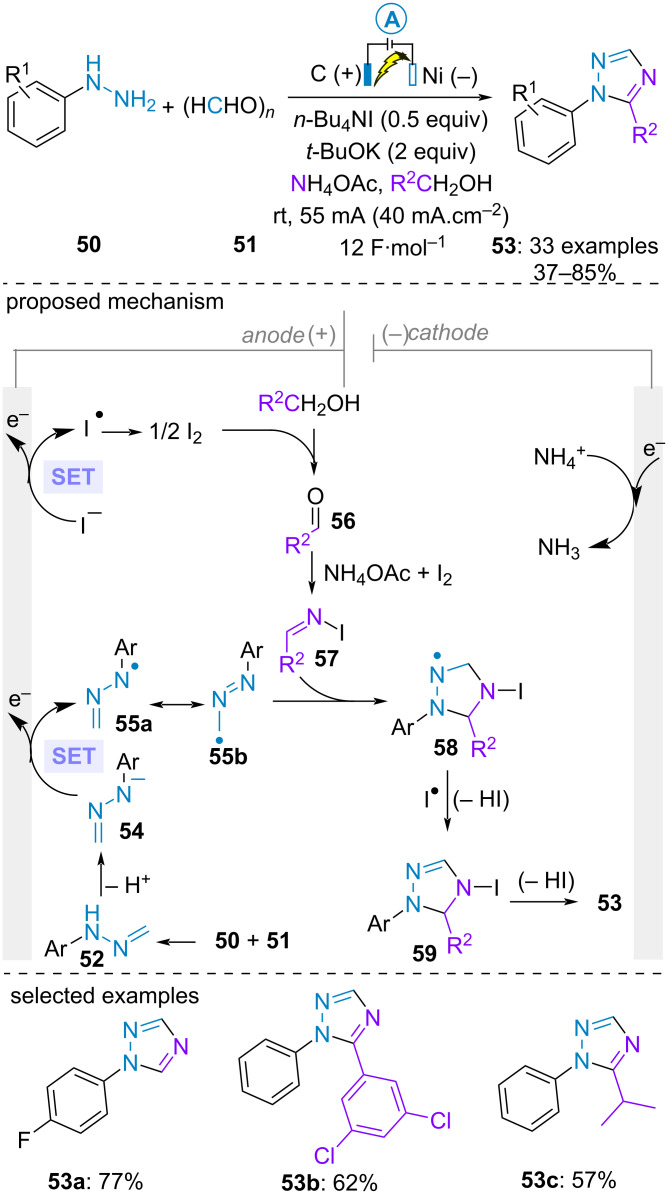
Synthesis of 1-aryl and 1,5-disubstitued 1,2,4-triazoles [49].

For the construction of non-ionic azacycle from aldehyde-derived *NH*-arylhydrazones, the electrolysis is generally conducted in the presence of iodide anion as electromediator. An important challenge is to avoid the competitive formation of the corresponding methoxy(phenylazo)alkane [46–47] or carbonyl compound [48] upon SET anodic oxidation of the hydrazone and subsequent reaction with methanol or water, respectively. In 2018, Yuan and Yang developed a multicomponent synthesis of 1-aryl and 1,5-disubstitued 1,2,4-triazoles using tetrabutylammonium iodide as electromeditor [49]. 1-Methylene-2-arylhydrazine **52** was in situ generated through the condensation of paraformaldehyde **51** with arylhydrazines **50**, while the electrolyte ammonium acetate and the alcoholic solvent acted as N and C1 sources, respectively. The authors proposed the mechanism depicted in Scheme 10. The deprotonation of hydrazone **52** afforded anion **54** which underwent SET anodic oxidation to form radical **55**. In parallel, an elusive oxidation of alcohol electromediated by iodine would furnish aldehyde **56**. Electrogenerated iodine would further assist the reaction with ammonia to form *N*-iodo aldimine intermediate **57**. Subsequent radical cycloaddition between **56** and **57** would furnish cyclic hydrazinyl radical **58**. Finally, the triazole would be obtained after hydrogen atom abstraction by iodide radical and deprotonation.

**Scheme 11:**
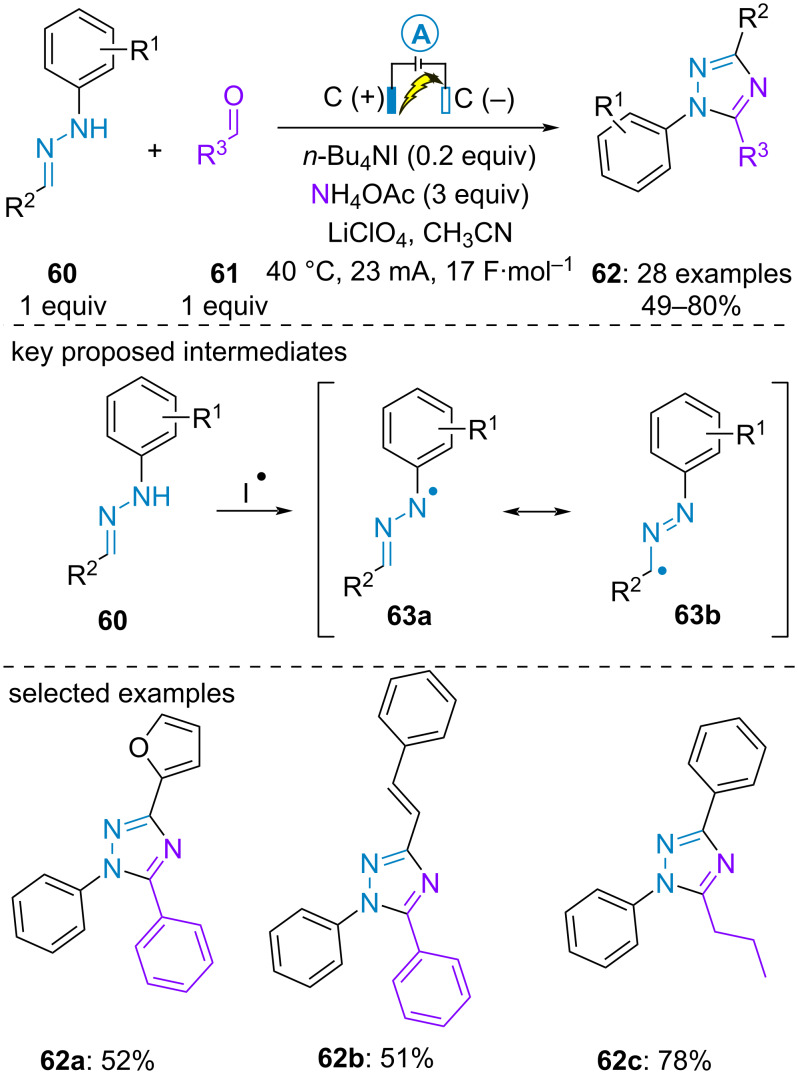
Synthesis of 1,3,5-trisubstituted 1,2,4-triazoles [50].

Inspired by these works, Li and Gu et al. proposed the electrosynthesis of 1,3,5-trisubstituted 1,2,4-triazoles from preformed aldehyde-derived *N*-arylhydrazones **60**, aldehydes **61** and ammonium acetate. Herein, the authors suggested the electromediated oxidation of *N*-arylhydrazone by electrogenerated iodide radical (Scheme 11) [50].

**Scheme 12:**
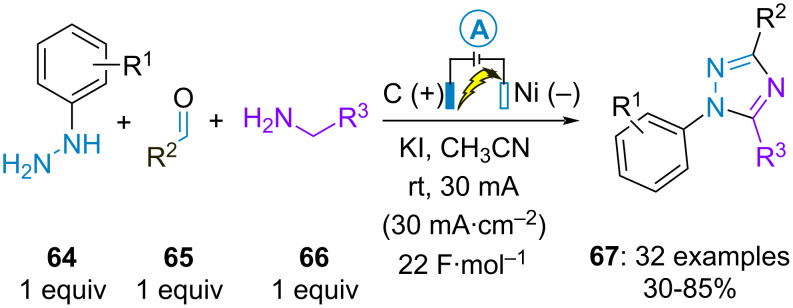
Alternative synthesis of 1,3,5-trisubstituted 1,2,4-triazoles [51].

In parallel, the group of Yuan achieved the electrosynthesis of 1,3,5-trisubstituted 1,2,4-triazoles **67** from arylhydrazines **64**, aldehydes **65** and primary amines **66**. Iodine-mediated electrooxidation of in situ-generated aldehyde-derived hydrazones was also proposed as key initial step of the transformation (Scheme 12) [51]. Concurrently, similar formal electrochemical cycloaddition reactions between preformed aldehyde-derived hydrazones and aliphatic amines were investigated by the groups of D. Tang [52] and Pan [53]. The latter employed ferrocene as electrocatalyst instead of iodide salts. Additionally, Tang et al. demonstrated that amidines could react with preformed aldehydes-derived hydrazones to produce similar 1,3,5-trisubstituted 1,2,4-triazoles [54].

**Scheme 13:**
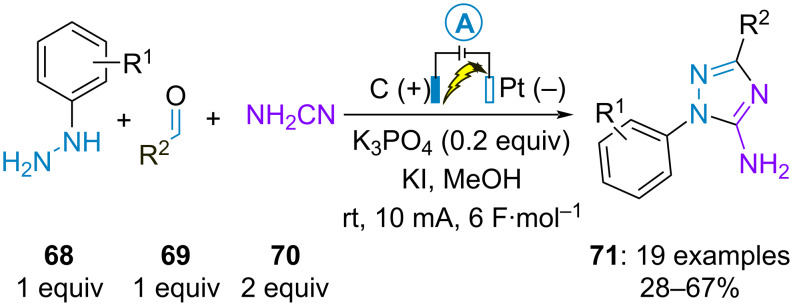
Synthesis of 5-amino 1,2,4-triazoles [55].

The last example of electrochemical synthesis of trisubstituted 1,2,4-triazoles was accomplished by D. Tang et al. through the formal cycloaddition between in situ-generated aldehyde-derived hydrazones and cyanoamine (**70**). The 5-amino derivatives **71** were obtained in moderate to good yields by employing potassium iodide as electrocatalyst (Scheme 13) [55]. Herein, the electrochemical approach constitutes an advantageous alternative to previous methods, which required the preparation of difficult-to-handle hydrazonoyl halides for the synthesis of 1-aryl-5-amino-1,2,4-triazoles from cyanamide [56–57].

**Scheme 14:**
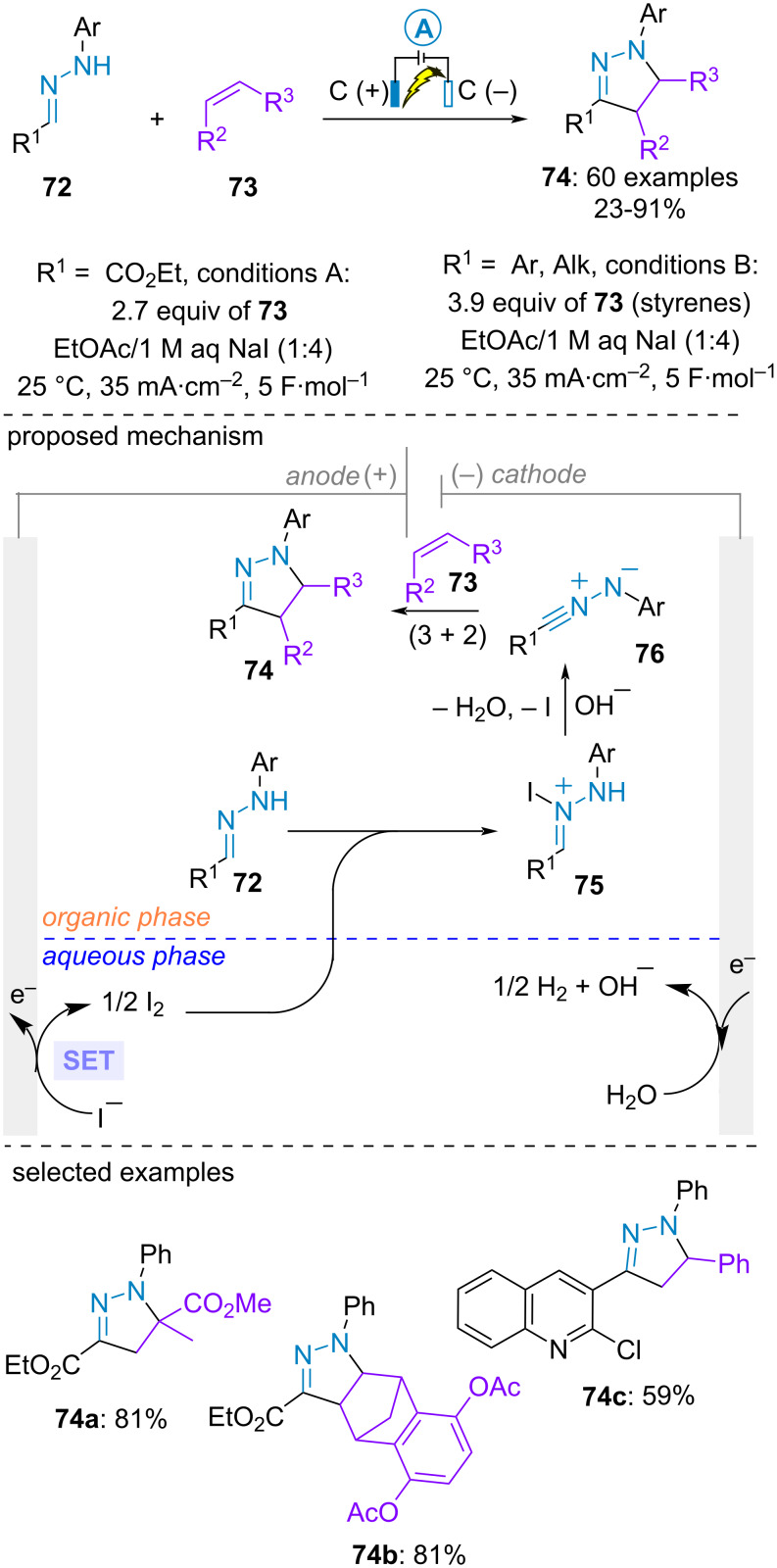
Synthesis of 1-arylpyrazolines [58].

Pyrazolines are highly important heterocyclic motifs in pharmaceutical and agrochemical industries. The (3 + 2)-cycloaddition between nitrile imines and alkenes represents one of the most efficient strategies to prepare these azacycles. However, conventional methods for the generation of the nitrile imine involved the use of unstable hydrazonoyl halides or the oxidation of aldehyde-derived hydrazones under harsh reaction conditions. In 2023, the group of Waldvogel presented a formal electrooxidative (3 + 2)-cycloaddition between aldehyde-derived hydrazones **72** and alkenes **73** to yield a large range of *N*-arylpyrazolines **74** under mild reaction conditions (Scheme 14) [58]. A biphasic system (aqueous/organic) was engineered during which the oxidation of the iodide mediator took place in the aqueous place, thereby protecting sensitive dipolarophiles such as styrenes from side reactions (e.g., overoxidation or polymerisation) in the organic phase. Ethyl acetate was employed as organic solvent for ethyl glyoxylate phenylhydrazone **72a** while methyl *tert*-butyl ether was preferred for aromatic and aliphatic aldehyde-derived hydrazones **72b**. Styrenes, enamines as well as electron poor aliphatic alkenes were all suitable dipolarophiles. From a mechanistic point of view, the authors proposed the electrogeneration of iodine in the aqueous phase. Under high stirring, the latter would react with *NH*-arylhydrazones **72** in the organic phase to furnish the *N*-iodo hydrazonium **75** and ultimately the nitrile imine **76** under basic conditions provided by the cathodic process. The critical role of the in situ cathodic generation of the base was supported by a control experiment in a divided cell, where no conversion was achieved. Final formal (3 + 2)-cycloaddition with the dipolarophiles **73** delivered the pyrazolines **74**. It is interesting to note that a complementary (3 + 2)-cycloaddition between aldehyde hydrazones and alkenes for the preparation of pyrazolines was proposed by Tong, Song, et al., achieving similar efficiency using oxone/KBr as an environmentally friendly oxidative system [59].

**Scheme 15:**
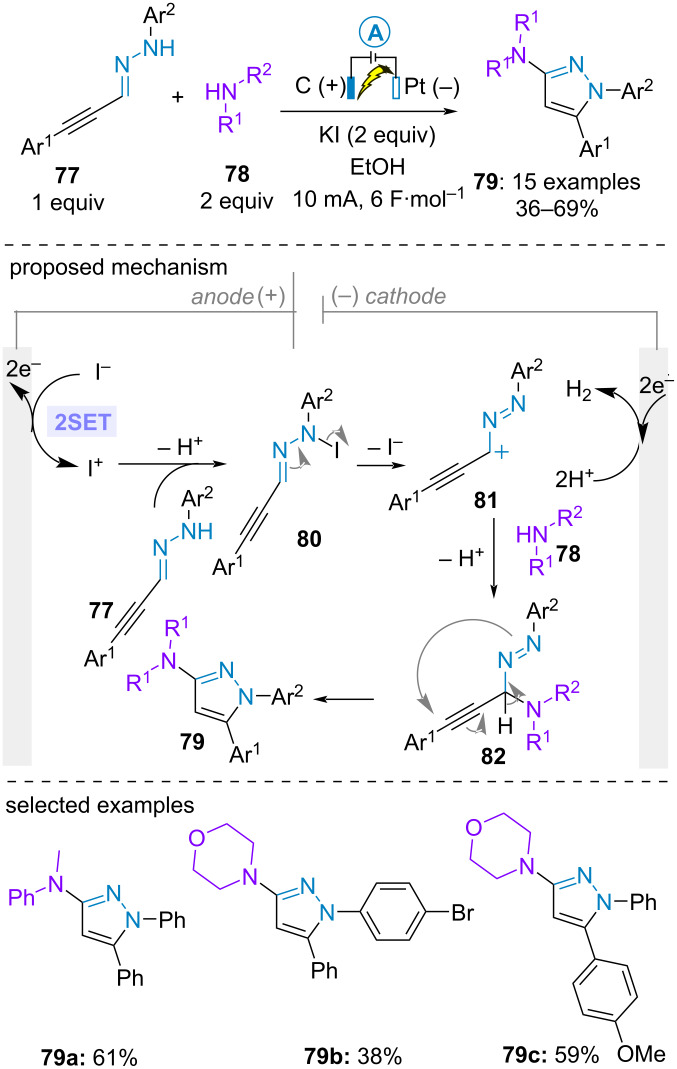
Synthesis of 3‑aminopyrazoles [60].

Most recently, Zhou and Ma et al. described the electrochemical access to 3‑aminopyrazoles **79** from arylpropynal-derived *NH*-arylhydrazones **77** and secondary amines **78** in moderate to good yields. Potassium iodide was employed as electrolyte and mediator in ethanol in an undivided cell equipped with a carbon graphite and a platinium cathode. The transformation initiated with the electrochemical generation of iodonium through a two-electron process. Subsequent reaction with the hydrazone and deprotonation formed the *N*-iodo hydrazone intermediate **80**, triggering the reaction with the amine **78** through cationic species **81**. Final cyclization delivered the desired pyrazole **79** (Scheme 15) [60].

**Scheme 16:**
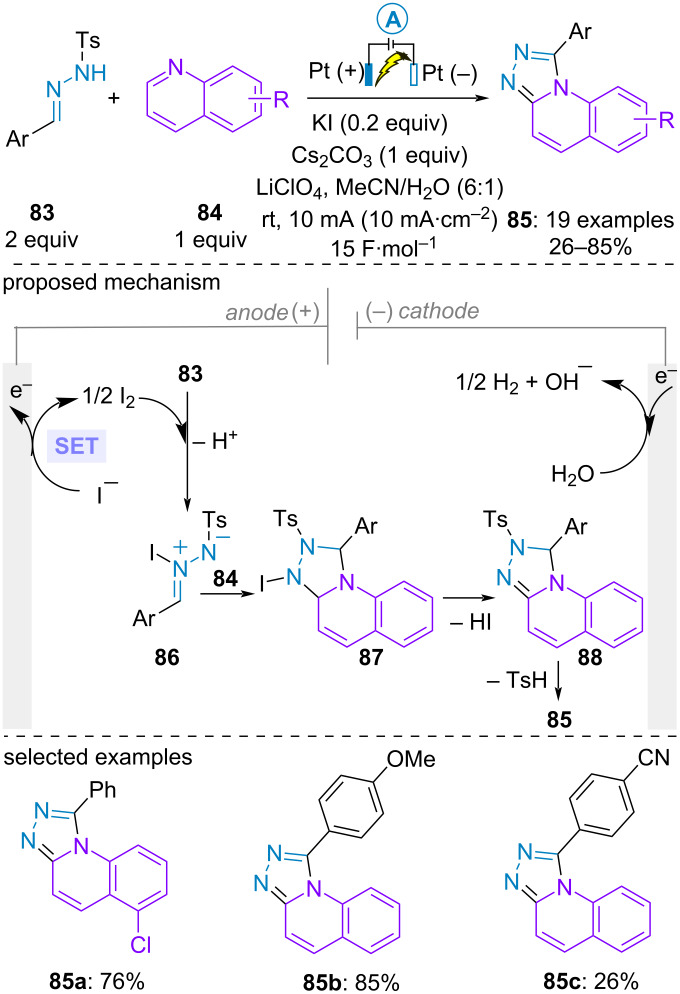
Synthesis of [1,2,4]triazolo[4,3-*a*]quinolines [61].·

The group of D. Tang engaged aromatic aldehyde-derived *NH*-tosylhydrazones **83** in an iodide-catalyzed electrochemical formal (3 + 2)-cycloaddition with quinolines **84** to build [1,2,4]triazolo[4,3-*a*]quinoline derivatives **85**. Better yields were obtained with hydrazones bearing an electron-rich substituent on the aromatic ring. The reaction initiated with anodic formation of iodine. The latter would react with hydrazone **83** to form the zwitterionic species **86** under basic conditions. Subsequent formal (3 + 2)-cycloaddition with the quinoline **84** formed fused system **87** which underwent elimination of iodide and sulfinic acid to furnish the aromatic product **85**, thereby regenerating the catalyst (Scheme 16) [61].

**Scheme 17:**
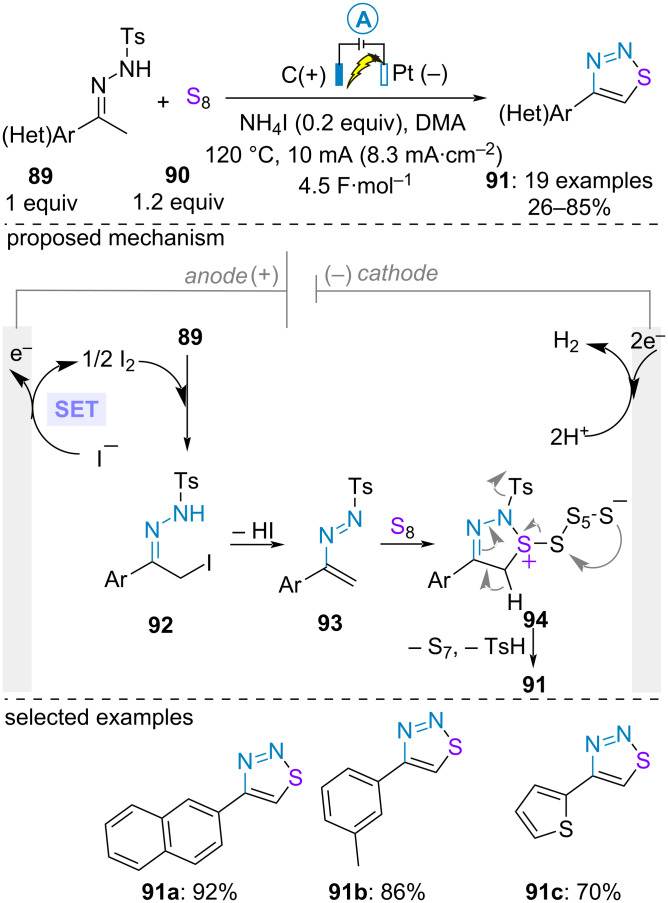
Synthesis of 1,2,3-thiadiazoles [64].

Given its abundance, stability, and low price, elemental sufur (S_8_) is an ideal source of sulfur atom to produce thiacycles [62]. In 2019, H.-T. Tang utilized this reagent in combination with (hetero)aromatic ketone-derived *NH*-tosylhydrazones **89** for the electrochemical construction of 1,2,3-thiadiazoles **91**. The electrolysis was conducted in an undivided cell at high temperature using ammonium iodide as electrocatalyst. Interestingly, no additional electrolyte was required. The transformation accommodated various substituents on the aromatic moiety regardless of their electronic properties. However, 4,5-disubstituted 1,2,3-thiadiazoles could not be accessed with this methodology. Mechanistically, control experiments with radical trapping agent such as TEMPO ((2,2,6,6-tetramethylpiperidin-1-yl)oxyl) or 1,1-diphenylethylene proceeded with similar efficiency, thereby excluding any radical pathway. As such, it was proposed that the catalytic electrooxidation of iodide (*E*_NH4I_ = 0.41 V and 0.75 V vs Ag/AgCl in dimethylacetamide for I^−^/I^3−^ and I^3−^/I_2_ redox couples) into iodine provided a green and mild tool to in situ generate the α-iodo ketone-derived *NH*-tosylhydrazone **92** and ultimately the azadiene **93** upon elimination of HI. In agreement with a previous report [63], further formal (4 + 1) cycloaddition with S_8_ followed by elimination of S_7_ and sulfinic acid gave rise to the aromatic cycle **91** (Scheme 17) [64].

**Scheme 18:**
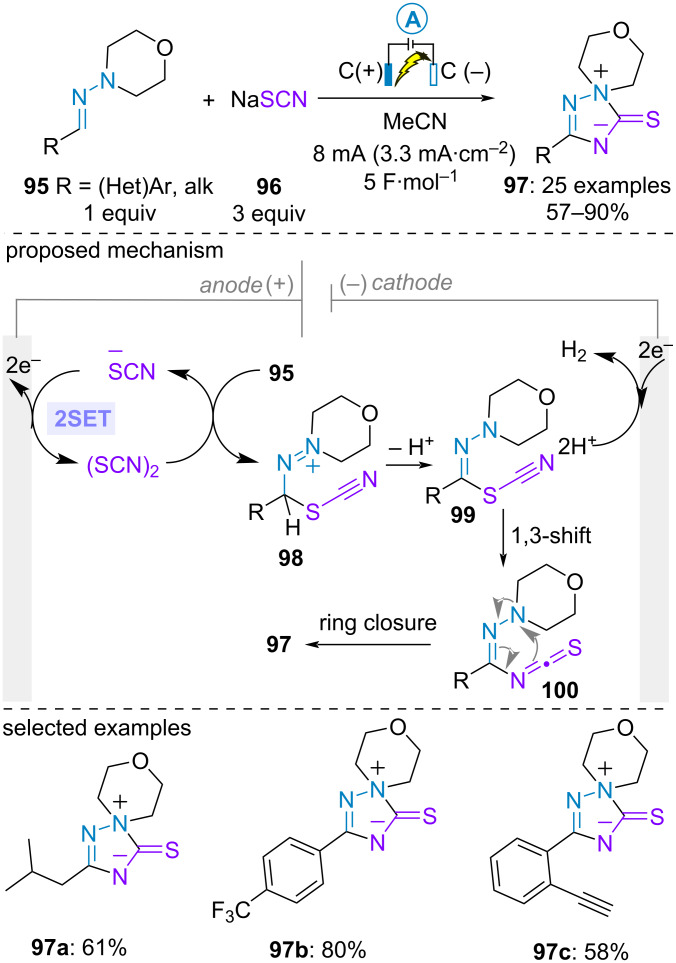
Synthesis of 5-thioxo-1,2,4-triazolium inner salts [65].

The direct C(sp^2^)–H functionalization of aldehyde-derived *N,N*-dialkylhydrazones has been also explored for constructing various azacycles. In 2021, the group of Ruan and Feng studied the electrochemical C(sp^2^)–H thiocyanation of aldehyde-derived *N*,*N*-dialkylhydrazones **95** with sodium thiocyanate (**96**) to prepare 5-thioxo-1,2,4-triazolium inner salts **97**. The electrolysis was conducted with two inexpensive graphite electrodes in the absence of additional supporting electrolyte. It could be easily performed on a gram scale, albeit with a slight decrease in the efficiency. It was compatible with a large array of aromatic aldehyde-derived hydrazones regardless of their electronic properties as well as aliphatic-aldehyde derived hydrazones. Cyclic voltammetry analysis showed that thiocyanate salt **96** possesses a lower oxidation potential than the hydrazone **95**. As such, the authors proposed the anodic generation of electrophilic thiocyanogen as the initial step. Further reaction with the hydrazone **95** and deprotonation led to hydrazonoyl thiocyanate intermediate **99**, which isomerized to the thermodynamically more stable isothiocyanate derivatives **100** through 1,3-shift. The latter underwent spontaneous ring closure to furnish the 1,2,4-triazolium **97** (Scheme 18) [65].

**Scheme 19:**
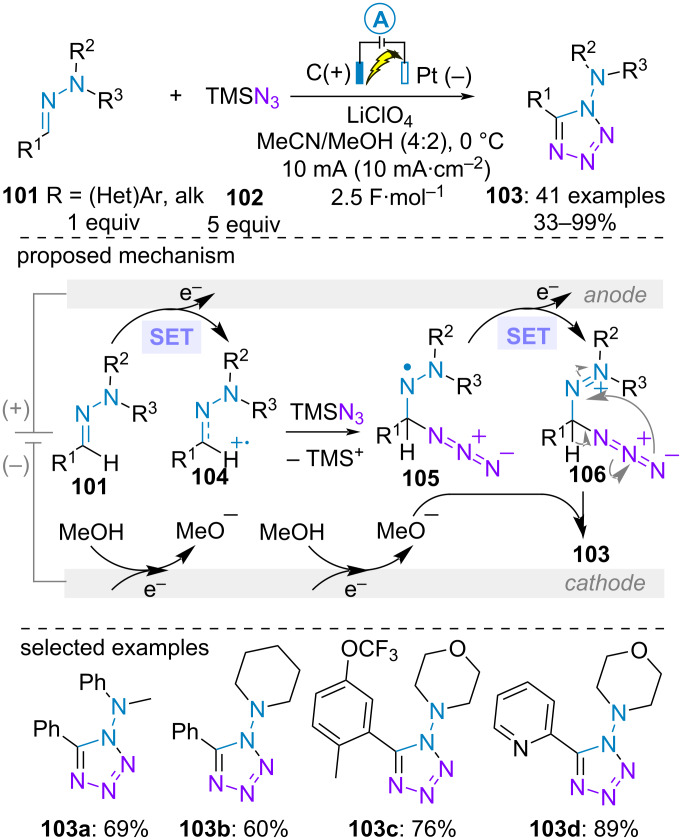
Synthesis of 1-aminotetrazoles [66].

In the last example of this section, only one nitrogen atom of the hydrazone takes part in the cycloaddition reaction. In 2018, Zhang et al. realized the electrochemical (3 + 2)-cycloaddition of trimethylsilyl azide (**102**) with aldehyde-derived *N,N*-disubstituted hydazones **101** for synthesiszing tetrazoles **103**. Sodium azide could be an alternative source of azide, albeit with both slightly lower yields and Faradic efficiency. Remarkably, (hetero)aromatic as well as aliphatic aldehydes proved to be efficient. Various substituents on the N(sp^3^) atom were well tolerated and the best result was obtained with a morpholine ring. Based on cyclic voltammetry studies, the transformation initiated with the anodic oxidation of hydrazone **101** to form highly electrophilic radical cationic species **104**. Subsequent addition of azide **102** and desilylation formed hydrazinyl radical **105** which underwent second anodic oxidation/cyclization and deprotonation sequence to build the 1-aminotetrazole **103** (Scheme 19) [66]. It is interesting to note that this mechanism differs from the one proposed by Zhu et al*.,* who reported a hypervalent iodide-mediated similar cycloaddition through the generation of azide radical, although with similar yields [67].

### Synthesis of functionalized hydrazones through C(sp^2^)–H functionalization of aldehyde-derived hydrazones

**Scheme 20:**
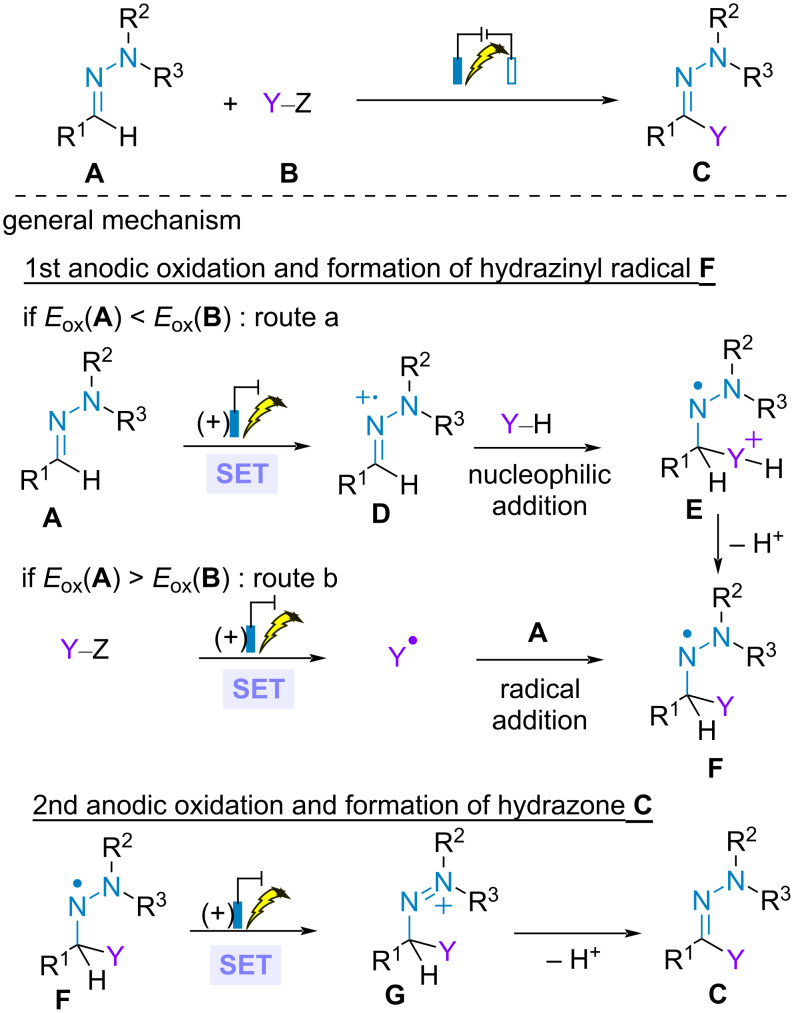
C(sp^2^)–H functionalization of aldehyde-derived hydrazones: general mechanisms.

The direct electrochemical C(sp^2^)–H functionalization of aldehyde-derived *N,N*-disubstituted hydrazones provides an efficient tool for accessing various hydrazones under mild reaction conditions and, to some extent, broadening the scope of more conventional radical approaches. Typically, two mechanistic pathways can be proposed depending on the oxidation potentials of each partner. If the hydrazone is the most readily species to be oxidized, initial SET anodic oxidation of the hydrazone furnishes the highly electrophilic radical cation species **D**, which undergo nucleophilic addition of the second partner and deprotonation to produce hydrazinyl radical **F** (route a). Alternatively, if the partner possesses a lower oxidation potential than the hydrazone, then the transformation initiates with the anodic generation of radical species Y^•^, that adds to the hydrazone leading ultimately to hydrazinyl radical **F** as well (route b). In both cases, a second SET anodic oxidation and deprotonation yields the functionalized hydrazone (Scheme 20).

#### Initial SET oxidation of the hydrazone

**Scheme 21:**
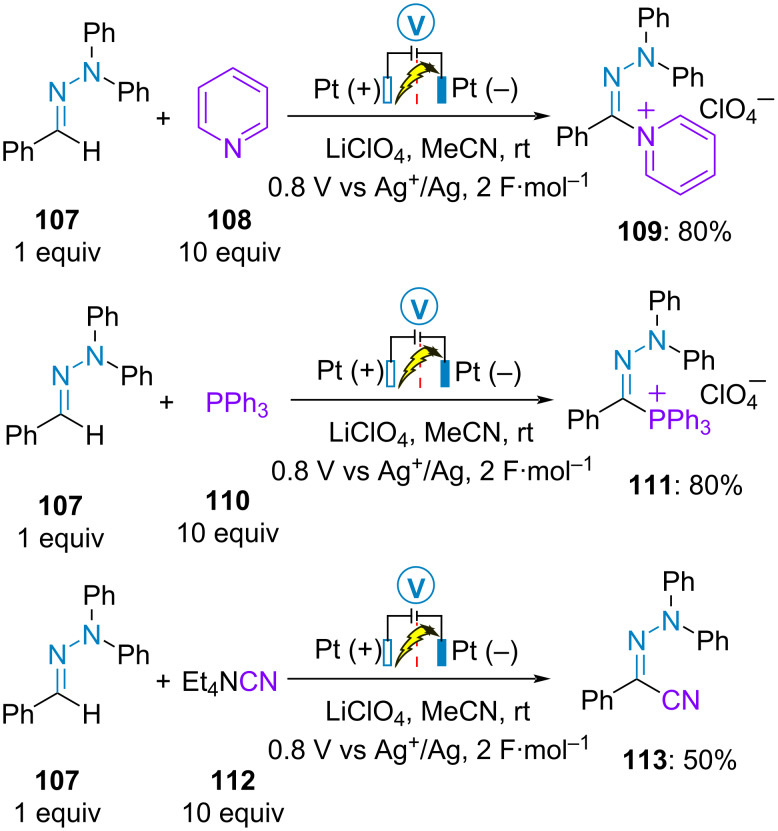
C(sp^2^)–H functionalization of benzaldehyde diphenyl hydrazone [68–69].

In 1973, Barbey and Caullet have shown that the benzaldehyde-derived *N,N*-diphenylhydrazone **107** dimerized upon electrochemical oxidation on platinum electrode [68]. The following year, they demonstrated that in the presence of an excess of pyridine, triphenylphosphine or tetraethylammonium cyanide, the corresponding pyridium **109**, phosphonium **111** and cyano hydrazones **113** were obtained, respectively (Scheme 21) [69].

**Scheme 22:**
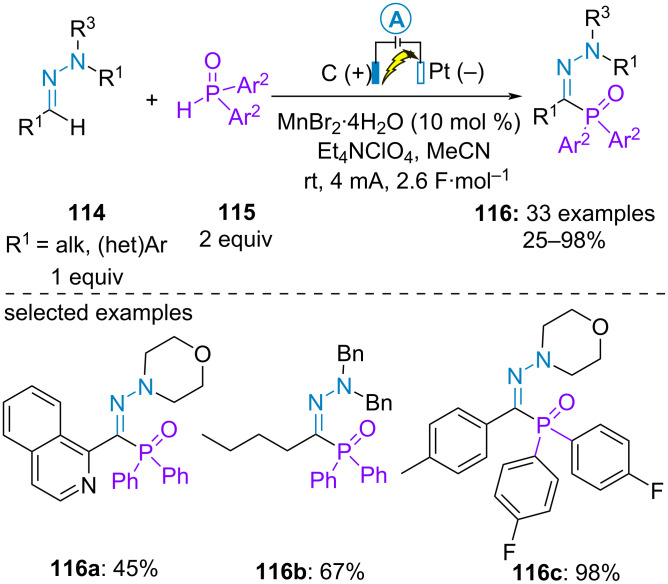
Phosphorylation of aldehyde-derived hydrazones [70].

In 2020, Ruan and Sun et al*.* communicated the electrochemical dehydrogenative coupling between (hetero)aromatic or aliphatic aldehyde-derived hydrazones **114** and diarylphosphine oxide **115**. The phosphorylation proceeded with slightly higher yield in the presence of a catalytic amount of manganese dibromide but its role was not clearly identified. Cyclic voltammetry analysis supported the initial oxidation of the hydrazone (*E*_p/2_ of 4-methylbenzaldehyde-derived hydrazone = 0.89 V vs Ag/AgCl in CH_3_CN and *E*_p/2_ of diphenylphosphine oxide > 0.89 V vs Ag/AgCl in CH_3_CN) (Scheme 22) [70]. Similar yields were previously obtained for the same transformation using a substoichiometric amount of potassium persulfate as oxidant [71].

**Scheme 23:**
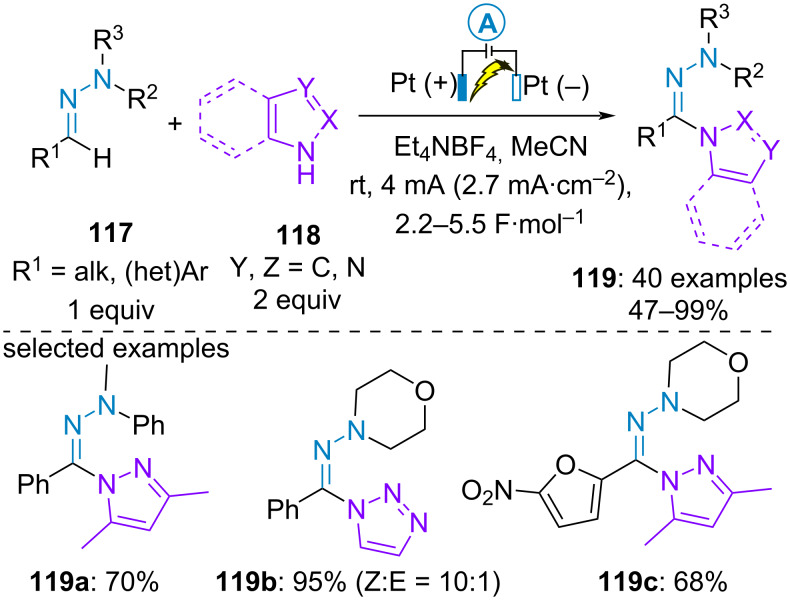
Azolation of aldehyde-derived hydrazones [72].

In 2022, the group of Huang employed aromatic azoles **118** as additional suitable nucleophilic partners for C(sp^2^)–H functionalization of aldehyde-derived *N,N*-disubstituted hydrazones **117**. A wide range of aldehyde-derived hydrazones reacted smoothly with various azoles including pyrazole derivatives, imidazole, pyrazole and triazoles (Scheme 23) [72]. Cyclic voltammetry studies confirmed that the azoles were redox inactive in the scan window while benzaldehyde-derived morpholino hydrazone could be readily oxidized at 1.18 V vs SCE in CH_3_CN supporting the route (a) of the general mechanism in Scheme 20.

#### Initial SET oxidation of the partner

**Scheme 24:**
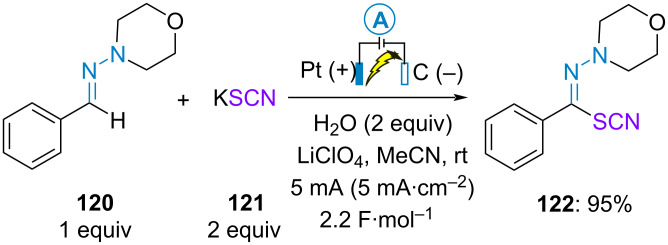
Thiocyanation of benzaldehyde-derived hydrazone **122** [73].

As mentioned above, if the partner is more readily oxidized than the hydrazone, then the general mechanism of the process is as described in Scheme 20, following route (b) for the first anodic oxidation. While investigating the electrochemical oxidative C(sp^2^)–H thiocyanation of ketene dithioacetals, Wang and Yang et al. mentioned one example of thiocyanation of benzaldehyde-derived hydrazone **120** using potassium thiocyanate as a source of thiocyanate radical. The electrolysis was conducted in the presence of two equivalents of water in acetonitrile with lithium perchlorate as electrolyte. It is interesting to notice that the isomerization of the product and subsequent ring closure discussed above (see Scheme 17) did not occur under these conditions (Scheme 24) [73].

**Scheme 25:**
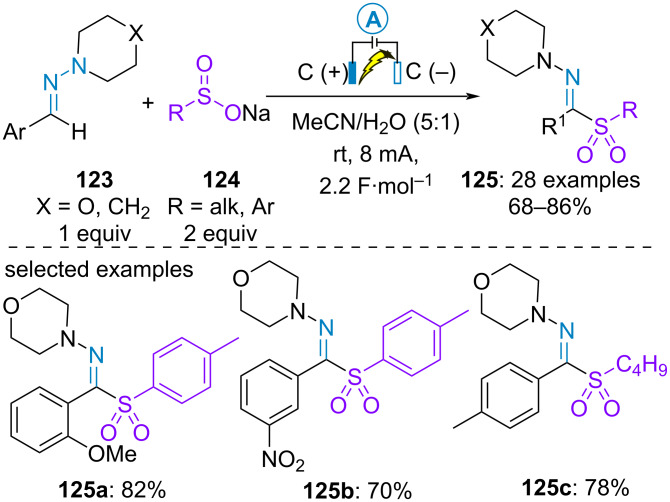
Sulfonylation of aromatic aldehyde-derived hydrazones [74].

In 2023, Hajra et al. provided an electrochemical method for the C(sp^2^)–H sulfonylation of aromatic aldehyde-derived *N,N*-dialkylhydrazones **123** under constant current with two carbon electrodes. Aromatic and aliphatic sodium sulfinates **124** were employed as sources of sulfinate radicals under electrolyte-free reaction conditions. The targeted product was obtained in high yield regardless of the electronic properties of the substituents on the aromatic ring of either the hydrazone or the sulfinate salt (Scheme 25) [74]. In parallel, a similar transformation was developed by Guo and Yang et al. Using two platinum electrodes and *n*-Bu_4_NBF_4_ as supporting electrolyte in a 1:1 mixture of acetonitrile/water, heteroromatic aldehyde-derived hydrazones as well as heteroaromatic sodium sulfinate salts were additional compatible reagents [75].

**Scheme 26:**
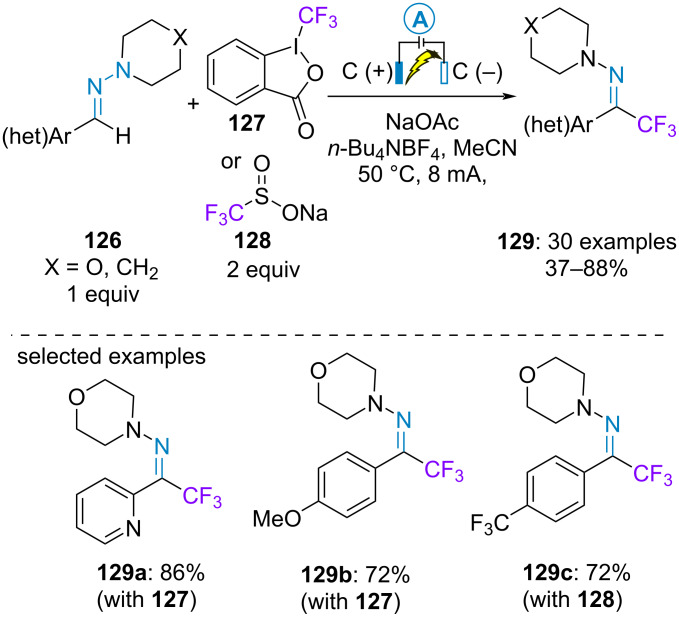
Trifluoromethylation of aromatic aldehyde-derived hydrazones [76].

In 2022, Xie et al. achieved the electrochemical C(sp^2^)–H trifluoromethylation of (hetero)aromatic aldehyde-derived hydrazones. The galvanostatic electrolysis was performed with two carbone graphite electrodes. The Togni reagent **127** was chosen as a source of trifluoromethyl radical with redox neutral and electron-rich hydrazones through a paired electrolysis. In contrast, the Langlois reagent **128** was preferred for electron-poor substrates through an electrooxidative process (Scheme 26) [76].

### Electrochemical access to diazo compounds

Diazo compounds are highly useful synthetic reagents in organic chemistry. For instance, they are regularly utilized as 1,3-dipoles in cycloaddition reactions or as precursor of carbenes under thermal, photochemical, and catalytic transition-metal-based transformations. However, their use is significantly hampered by their inherent instability and hazardous nature. Therefore, it is endlessly highly demanding to find a new mode of generation of diazo compounds from stable and safe precursors. The third part of this review is dedicated to electrochemical synthesis of diazo compounds from hydrazones. Transformations involving diazo compounds as either products or intermediates are covered.

**Scheme 27:**
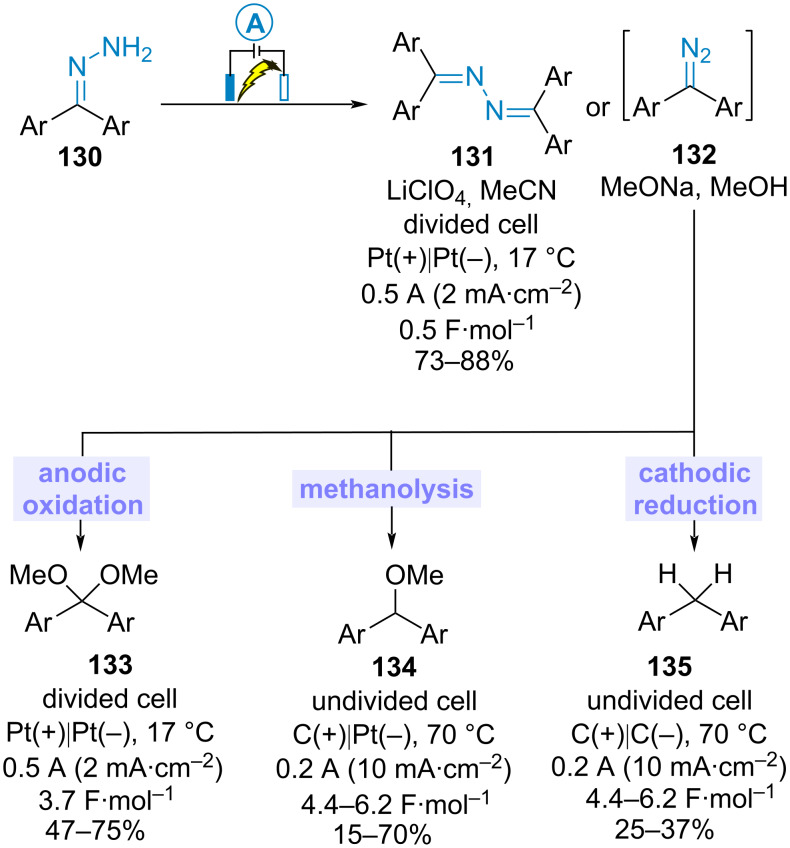
Electrooxidation of benzophenone hydrazones [77].

While investigating the electrochemical oxidation of benzophenone hydrazones **130**, Chiba et al. discovered that several products were obtained depending on the reaction conditions. For instance, the use of lithium perchlorate as supporting electrolyte in acetonitrile at room temperature in a divided cell equipped with two platinum electrodes led exclusively to diazines **131** in good yields. In contrast, when the electrolysis was conducted in methanol containing sodium methoxide in an undivided cell, three main products (namely benzophenone dimethyl acetals **133**, diphenylmethyl methyl ethers **134** and diphenylmethanes **135**) were obtained, the ratio of which was influenced by the electrode materials, the temperature and the amount of sodium methoxide. The range of isolated yields of the major products obtained under the appropriate reaction conditions is shown in Scheme 27. Based on control experiments, the authors proposed that **133**–**135** came from diazo intermediates **132**, which could not be isolated [77].

**Scheme 28:**
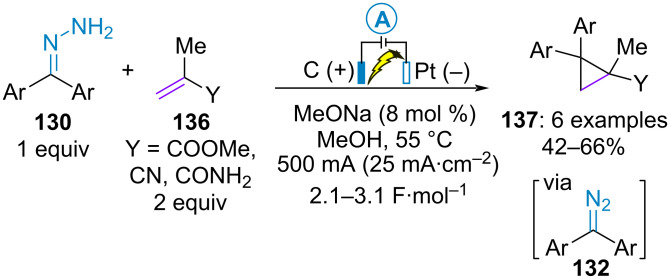
Electrooxidative coupling of benzophenone hydrazones and alkenes [77].

Building on this study and considering the high ability of diazo compounds to react with alkenes, Chiba et al. developed the electrochemical construction of diphenylcyclopropanes **137** from benzophenone hydrazones **130** and methacrylic acid derivatives **136**. The transformation was conducted on a large scale (60 mmol) in an undivided cell using graphite anode and platinum cathode materials at 55 °C with a minimal amount of sodium methoxide, thereby limiting side products formation such as **133**–**135** or some oligomers of ethylenes (Scheme 28) [77].

**Scheme 29:**
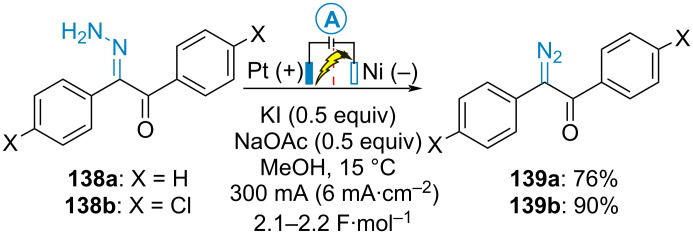
Electrosynthesis of α-diazoketones [78].

In 2002, Okimoto et al. reported the indirect electrochemical oxidation of benzilhydrazones **138** using potassium iodide as electromediator for synthesising α-diazoketones **139** in good yields. The electrolysis was performed at low temperature in a divided cell equipped with a platinum anode and a nickel cathode. It was not possible to isolate the diazo compounds arising from the electrooxidation of 4,4’-dimethylbenzilhydrazone and 4,4’-dimethoxybenzilhydrazone (X = H, Cl). In these cases, the diazo compounds directly underwent both Wolff rearrangement and overoxidations to yield a mixture of the corresponding diphenyl acetal and benzil dimethyl acetal (akin to the conversion of **132** into **133** in Scheme 27), respectively (Scheme 29) [78]. Using a similar procedure, the same authors subsequently reported the electrochemical oxidation of dibenzoylbenzene dihydrazones into the corresponding bisdiazo compounds [79].

**Scheme 30:**
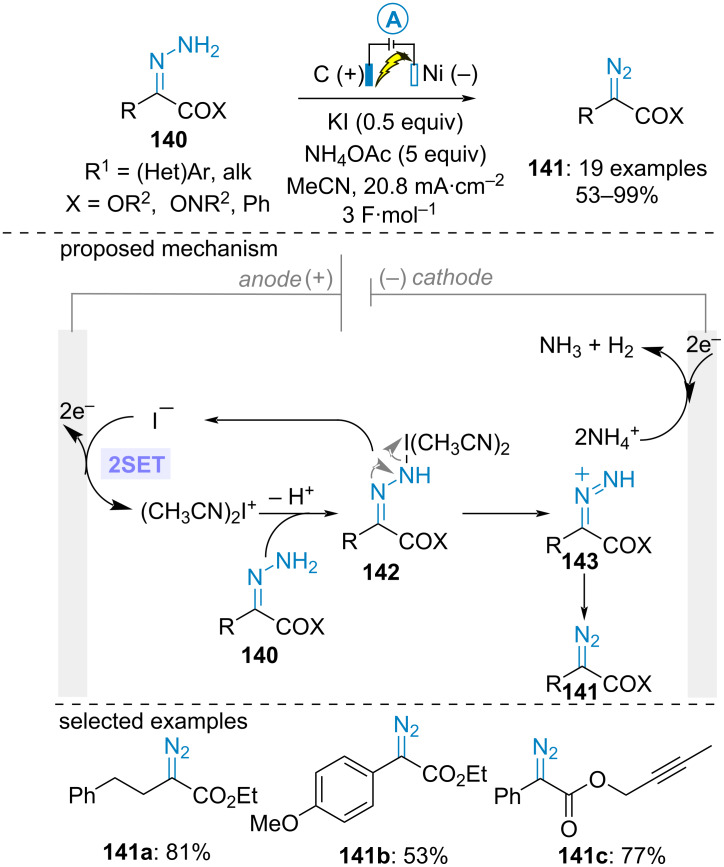
Electrosynthesis of stable diazo compounds [80].

In 2022, Ollevier and Lam et al. undertook an in-depth study for accessing stabilized diazo compounds through anodic oxidation of simple unsubstituted hydrazones **140**. After intensive parameter optimizations, good yields were achieved under galvanostatic conditions in a simple divided cell equipped with cheap carbon graphite anode and nickel cathode using potassium iodide and ammonium acetate as electrolyte. Cyclic voltammetry analysis suggested that potassium iodide would serve as an electromediator to oxidize the hydrazone through an inner-sphere electron transfer. Moreover, the electrolysis proceeded more efficiently in the presence of ammonium acetate since (i) the acetate anion would serve as a base to facilitate the oxidation of the hydrazone and (ii) the ammonium cation would provide a performant proton source to counterbalance the overall process, thereby protecting the diazo product from cathodic reduction (akin to the conversion of **132** into **135** in Scheme 27). Based on these studies, the authors proposed the initial anodic formation of the iodonium species [(CH_3_CN)_2_I^+^], which would react with the hydrazone to furnish the protonated diazo compound **143** via the *N*-iodo hydrazone intermediate **142**. Further acetate anion-assisted deprotonation ultimately led to the desired diazo compounds **141** (Scheme 30) [80]. A nice application of this procedure involved the (3 + 2)-cycloaddition reaction between electrogenerated diphenyldiazomethane and methyl acrylate delivering the corresponding pyrazoline in good yield (68%).

**Scheme 31:**
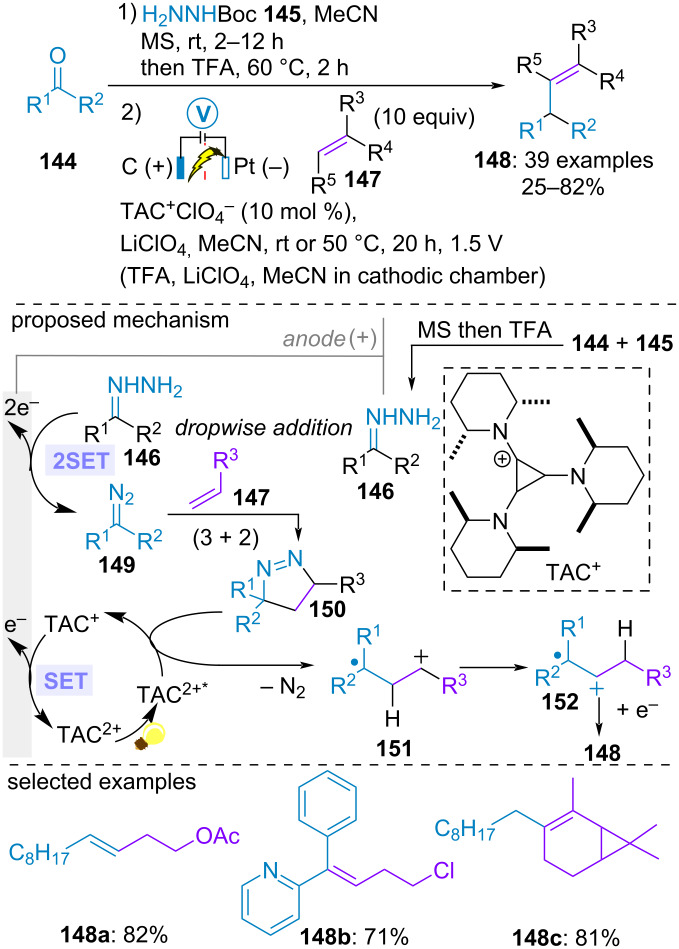
Photoelectrochemical synthesis of alkenes through in situ generation of diazo compounds [81].

Olefins are highly common structural motifs in natural and synthetic organic compounds. They are also often employed as pivotal element during the total synthesis of natural products. Therefore, the development of mild and efficient methods to access this motif from readily available starting materials is a never-ending quest. With the aim to propose an alternative to the classical olefination of carbonyls through Wittig, Julia, Peterson and Tebbe-type reactions, the group of Lambert et al. implemented an elegant electrophotocatalytic carbonyl-olefin cross-coupling in an undivided cell equipped with a carbon felt anode and a platinum cathode in the presence of their own trisaminocyclopropenium cationic photocatalyst TAC^+^. The electrolysis was carried out at a constant cell potential of 1.5 V under white light irradiation. The carbonyl compound **144** was initially treated with *tert*-butyl carbazate (**145**) in acetonitrile in the presence of molecular sieves, followed by the addition of trifluoroacetic acid (TFA) to yield the unsubstituted hydrazone **146**. Dropwise addition of this solution during the electrolysis to the anodic chamber containing the olefin **147**, lithium perchlorate and the photocatalyst in acetonitrile delivered the desired olefin product **148**. From a mechanistic point of view, the photoelectrochemical transformation began with the anodic oxidation of the hydrazone **146** to the corresponding diazo compound **149**, akin to Lam and Ollevier’s work. Subsequent (3 + 2)-cycloaddition with the olefin partner **147** formed cyclic diazene **150**. Meanwhile, anodic oxidation of TAC^+^ generated photosensitive TAC^2+^ (*E*_1/2_(TAC^2+^/TAC^+^) = +1.3 V vs SCE). Subsequent SET between highly oxidizing photoexcited species TAC^2+*^ (E_1/2_(TAC^2+*^/TAC^+^) = +3.3 V vs SCE) and **150** generated distonic species **151** by denitrogenation. After Wagner–Merweein shift, the resulting radical cation **152** would undergo SET reduction from an electron donor to furnish the olefin product **148**. In the cathodic chamber, reduction of the acidic proton of TFA counterbalance the overall transformation. A wide range of carbonyl compounds including aromatic and aliphatic aldehydes and ketones as well as various alkene partners were compatible. Of note, the most thermodynamically stable distonic radical was formed, thereby allowing the formation of only one isomer of the olefin product (Scheme 31) [81].

### Miscellaneous

**Scheme 32:**
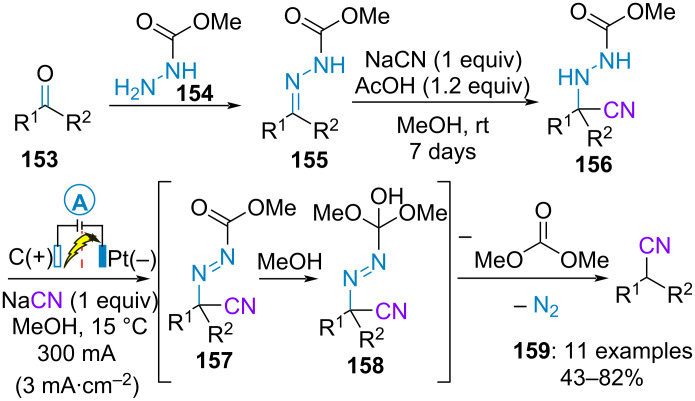
Synthesis of nitriles [82].

In 1990, Chiba and Okimoto established an electrosynthetic procedure to convert the carbonyl group of aliphatic ketones **153** into a cyanomethylene group. Condensation of **153** with methyl carbazate (**154**) furnished the corresponding methoxycarbonylhydrazone **155**. The latter was charged in the anolyte compartment of a divided cell containing sodium cyanide and acetic acid in methanol and was allowed to stand for several days in the dark in the absence of an electrical current, thus allowing the addition of in situ-generated HCN to the hydrazone **155**. After addition of a second equivalent of sodium cyanide, the resulting HCN adduct **156** was electrolyzed under constant current to form diazene **157**, which subsequently underwent addition of methanol/fragmentation and extrusion of nitrogen to yield the nitrile derivative **159**. The transformation proceeded neither with aldehydes nor with aromatic ketones (Scheme 32) [82].

**Scheme 33:**
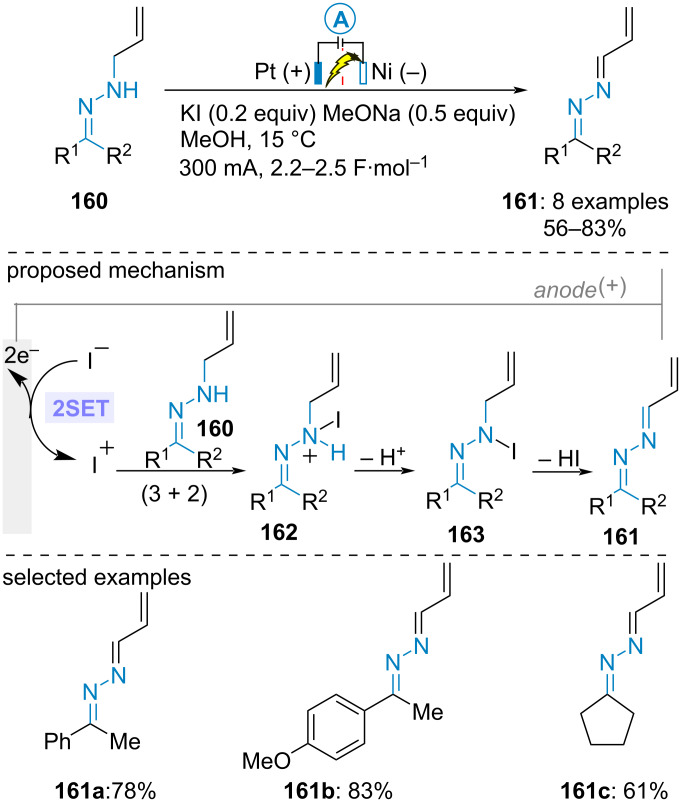
Electrochemical oxidation of ketone-derived *NH*-allylhydrazone [83].

In 2008, Okimoto et al. reported the electrochemical oxidation of ketone-derived *NH*-allylhydrazones **160** into the corresponding azines **161**. The electrolysis was conducted in a divided cell equipped with a nickel coil cathode and a platinum anode under constant current in methanol. Both potassium iodide and sodium methoxide were required as electrolyte supports to achieve high efficiency. The former would assist the oxidation of the hydrazone through the in situ generation of iodonium as oxidant while the latter would facilitate the deprotonation of iodoammonium **162** and the elimination of HI from *N*-iodo intermediate **163**. The best yields were obtained with aromatic ketone-derived hydrazones (Scheme 33) [83].

## Conclusion

Given the rich reactivity profile of hydrazones, the electrooxidative transformations of such a molecular building block provides a fascinating route to valuable compounds under mild and safe reaction conditions. Alone, the electrooxidation of *NH*-aryl, -tosyl, and -acylhydrazones triggered the cyclization of a radical cation intermediate enabling the construction of pyrazole, triazole and oxadiazole derivatives, while the electrooxidation of unprotected NH_2_ hydrazones constitutes a useful mean to access to relevant diazo compounds as products or synthetic intermediates. When coupled with a second reactant, electrooxidative processes gave rise to various azacycles or to functionalized hydrazones through C(sp^2^)−H functionalization of aldehyde-derived hydrazones. In both cases, transformations involving either the initial oxidation of the hydrazone or the partner have been demonstrated by carefully adapting the reaction conditions to selectively oxidize the desired compound. In some cases, the use of an electromediatior such as an iodide anion helped in achieving the desired selectivity. In line with the continuing increasing interest for both electrosynthetic organic transformations and the chemistry of hydrazones, coupling with other partners or examination of different hydrazones, such as trifluoromethylated hydrazones, should enable the straightforward access to diversely functionalized molecules. Hopefully, this review will stimulate further development in this area which could eventually find useful applications in industry.

## Data Availability

Data sharing is not applicable as no new data was generated or analyzed in this study.
